# The population prevalence of solitary confinement

**DOI:** 10.1126/sciadv.abj1928

**Published:** 2021-11-26

**Authors:** Hannah Pullen-Blasnik, Jessica T. Simes, Bruce Western

**Affiliations:** 1 Columbia University, New York, NY, USA.; 2 Boston University, Boston, MA, USA.

## Abstract

Solitary confinement is a severe form of incarceration closely associated with long-lasting psychological harm and poor post-release outcomes. Estimating the population prevalence, we find that 11% of all black men in Pennsylvania, born 1986 to 1989, were incarcerated in solitary confinement by age 32. Reflecting large racial disparities, the population prevalence is only 3.4% for Latinos and 1.4% for white men. About 9% of black men in the state cohort were held in solitary for more than 15 consecutive days, violating the United Nations standards for minimum treatment of incarcerated people. Nearly 1 in 100 black men experienced solitary for a year or longer by age 32. Racial disparities are similar for women, but rates are lower. A decomposition shows that black men’s high risk of solitary confinement stems primarily from their high imprisonment rate. Findings suggest that harsh conditions of U.S. incarceration have population-level effects on black men’s well-being.

## Teaser

One in 9 black men in Pennsylvania has been held in solitary confinement by age 32 and 1 in 100 for a year or more.

## INTRODUCTION

Black men in the United States are imprisoned at disproportionately high rates. As the U.S. incarceration rate grew to historically high levels in the early 2000s, this disparity has resulted in population level effects. Data from the 1970s to 2000s indicate that 20 to 30% of black men have been to prison by their mid-30s (*1*, *2*). Although period prevalence estimates describe the broad extent of incarceration, they convey little about prison conditions or racial disparities in the severity of incarceration.

Solitary confinement involves intense isolation that differs substantially from the experience of incarceration in the general prison population. Individuals are typically locked in their cells for 22 or 23 hours each day. Meals and toilet use take place inside the cell with only an hour outside for, say, recreation or showers. Access to rehabilitation programs, recreation activities, medical appointments, commissary supplies, phone calls, and visitation is severely restricted (*3*–*5*). Although conditions of solitary confinement vary across prisons and jurisdictions, three characteristics have come to define its practice in the United States: 22- or 23-hour confinement in a cell each day, severe restrictions on prison activities such as visits or programming, and widespread use of long-term isolation (*3*–*5*). There are few detailed analyses of the prevalence of solitary confinement, but national surveys providing point-in-time estimates indicate that about 4% of the state prison population is held in solitary confinement on an average day (*3*, *5*, *6*). Researchers also report racial disparities reflecting high rates of solitary confinement among incarcerated black and Latino people (*7*).

The official purposes of solitary confinement are typically divided into punishment and prison management. As punishment, sometimes called disciplinary custody, prison authorities use solitary confinement as a response to misconduct charges such as fighting or drug use. For prison management, often called administrative custody, authorities may use solitary confinement to separate those deemed to pose a threat to staff or other incarcerated people or as protective custody for those who feel or are determined to be unsafe in the general prison population. Although the purposes of solitary confinement vary, prison conditions and restrictions are often similar whether incarcerated in disciplinary or administrative custody (*3*).

Solitary confinement has been found to have a variety of negative effects. Much of the research has focused on mental health and the harm experienced by incarcerated people with preexisting mental illness (*8*–*10*). Evidence for negative mental health effects is consistent with high rates of suicidality among those with histories of solitary confinement (*11*). After prison, people who have been incarcerated in solitary confinement also experience higher risks of new criminal convictions, unemployment, and mortality (*12*–*14*).

The most harmful effects of solitary confinement have been reported for long periods of extreme isolation (*15*). Extended solitary confinement has been found to be especially harmful to mental health, associated with anxiety, depression, impulse control disorder, social withdrawal, lethargy, apathy, self-harming, and suicidal behavior (*15*). Infamous cases of injustice have also involved lengthy incarcerations in solitary confinement (*16*–*18*).

Acknowledging harms and threats to human rights accompanying sustained solitary confinement, the United Nations Standard Minimum Rules for the Treatment of Prisoners prohibited “prolonged solitary confinement” in excess of 15 consecutive days. Similar standards have recently been adopted in Colorado and New York (*19*, *20*). U.S. health organizations have opposed prolonged solitary confinement (*21*), focusing on the harms for those with serious mental illness (*22*). Federal courts also recognized the harms, finding certain forms of solitary confinement unconstitutional, notably for incarcerated people with mental illnesses (*23*–*26*).

Given evidence of harmful effects and racial disparity, what is the prevalence of solitary confinement in the general population for men and women in different racial and ethnic groups? This paper uses administrative data from the Pennsylvania Department of Corrections to estimate the population prevalence of imprisonment to age 30 and of solitary confinement to age 32 for men and women in four racial/ethnic groups: non-Latino white, non-Latino black, Latino, and any other race or ethnicity. Pennsylvania has the sixth largest prison population among all 50 states, and its incarceration rate and racial disparity in incarceration are approximately equal to the national average (*27*, *28*). Pennsylvania state prisons thus include a relatively large share of the U.S. prison population, and patterns of racial disparity resemble the U.S. pattern more broadly. We estimate the likelihood of having ever been imprisoned or held in solitary confinement from age 18 to 32 for a birth cohort born 1986 to 1989. To study prolonged isolation, we also estimate the likelihood by age 32 of being held in solitary confinement for up to a year or longer. These estimates of cumulative risk describe the prevalence of imprisonment and solitary confinement among Pennsylvania residents by their early 30s. We find evidence of large racial disparities, with black men far more likely to experience imprisonment, solitary confinement, and long periods of solitary confinement compared to other demographic groups. We decompose the disparity into components related to the disparity in imprisonment and the disparity in solitary confinement conditional on imprisonment. Estimates of the prevalence of solitary confinement for different racial and ethnic groups show how the pains of imprisonment are unequally distributed in the population and how imprisonment is disproportionately damaging for black and Latino communities.

## RESULTS

The current analysis uses administrative records that provide data on admissions to Pennsylvania prisons from 2007 until 2016 and admissions to solitary confinement until 2018. We follow a birth cohort, born 1986 to 1989, from age 18 until the oldest are aged 30 in 2016 (the final year for measuring imprisonment) and aged 32 in 2018 (the final year for measuring solitary confinement). We apply demographic life table methods to these data to calculate the risk of first-time imprisonment and solitary confinement at each year of age, adjusting for the effects of mortality and migration in the population (*29*, *30*). With estimates of the at-risk population experiencing imprisonment or solitary confinement each year, we calculate a cumulative risk that describes the proportion of the birth cohort that has ever experienced the event by a given age. We report estimates of the cumulative risks of imprisonment by age 30 and solitary confinement by age 32 for Pennsylvania men and women, born 1986 to 1989.

Table 1 describes the reasons for first-time solitary confinement provided in prison administrative records for men and women in the study birth cohort. The table reports administrative and disciplinary custody status for first-time solitary cases and the official misconduct charge issued by prison staff in cases of disciplinary custody. Around half of all incarcerated men and a third of women were first sent to solitary confinement not for officially charged misconduct but for administrative custody. For both men and women, rates of administrative custody were higher for white people and lower for black people. While prison misconduct charging can be arbitrary and unprotected by due process, official reasons for disciplinary custody in Pennsylvania were often not severe. Among those sent to solitary confinement for disciplinary custody, a minority were charged with violence by prison authorities. The most common categories of charged misconduct included refusal to follow the orders of prison staff (defiance), using abusive or obscene language (verbal threats), and possession of contraband such as drugs or weapons. Black men and women were more than twice as likely to be charged with verbal threats leading to solitary confinement as white men and women.

**Table 1.** First solitary charge. Percentage distribution of the recorded reasons for first-time solitary confinement for a Pennsylvania prison admission cohort, born 1986 to 1989, by gender, race, and ethnicity.

	**White (%)**	**Black (%)**	**Latino (%)**	**Other (%)**	**Total (%)**
*Men*					
Administrative custody	58.3	43.8	50.6	52.7	49.8
Disciplinary custody:*					
Violent misconduct	8.3	13.9	11.3	7.3	11.6
Verbal threat	7.7	19.0	12.3	9.1	14.1
Contraband	14.4	8.4	11.1	14.5	10.9
Defiance	11.3	15.0	14.8	16.4	13.7
Sample size (*N*)	3247	4706	959	55	8967
*Women*					
Administrative custody	44.5	25.0	12.8	12.5	35.9
Disciplinary custody:^*^					
Violent misconduct	11.0	25.0	10.3	12.5	15.2
Verbal threat	7.7	21.4	25.6	25.0	13.4
Contraband	15.1	8.3	7.7	0.0	12.3
Defiance	21.7	20.2	43.6	50.0	23.2
Sample size (*N*)	337	168	39	8	552

*In the case of multiple charges, the most severe charge is reported for disciplinary custody.

Table 2 shows the age-specific and cumulative risks of imprisonment to age 30 and solitary confinement to age 32 for all Pennsylvania residents in the study birth cohort. The cumulative risk describes the number of people ever imprisoned or placed in solitary confinement for at least 1 day as a proportion of the cohort population, adjusted for mortality and migration. The age-specific risk of first-time imprisonment for an individual in the 1986–1989 birth cohort peaks at age 22. By age 30, nearly 3% of the birth cohort in the state has been admitted to prison at least once. About half of those incarcerated are estimated to have been placed in solitary confinement at least once for at least 1 day. The age-specific risk of solitary confinement peaks at 24 years, about 2 years older than the age of peak imprisonment risk. We estimate that 1.5% of the state’s 1986–1989 birth cohort has been incarcerated in solitary confinement for at least 1 day by age 32.

**Table 2.** Life table results. Life table calculations for risk of incarceration by age 30 and solitary confinement by age 32, Pennsylvania (2007–2018).

**Age**	***Prison incarceration***		***Solitary confinement***	
	**Age-specific risk (%)**	**Cumulative risk (%)**	**Age-specific risk (%)**	**Cumulative risk (%)**
18	0.05	0.05	0.02	0.02
19	0.13	0.19	0.03	0.05
20	0.25	0.44	0.10	0.15
21	0.31	0.74	0.13	0.28
22	0.34	1.08	0.14	0.42
23	0.33	1.40	0.16	0.58
24	0.31	1.71	0.17	0.74
25	0.30	2.01	0.15	0.89
26	0.27	2.27	0.15	1.05
27	0.24	2.51	0.13	1.18
28	0.17	2.67	0.12	1.30
29	0.11	2.77	0.07	1.37
30	0.07	2.84	0.06	1.43
31	–	–	0.03	1.46
32	–	–	0.00	1.46

Estimates of the prevalence of imprisonment in Pennsylvania are similar to findings from national studies (*30*). Nearly one in five (19.1%) black men in Pennsylvania, born 1986 to 1989, has been imprisoned by age 30 compared to 6.6% of Latino men and fewer than 3% of white men (Table 3). The relative risk of imprisonment for black men is nearly seven times the risk for white men. Latino men experience about twice the cumulative risk of imprisonment as that estimated for white men.

**Table 3.** Cumulative risk by race. Cumulative risk of incarceration by age 30 and of solitary confinement by age 32 for Pennsylvania men and women born 1986 to 1989 by race and ethnicity. Risk ratios show the race-specific risk compared to the risk for white individuals.

	**Imprisonment by age 30 (%)**	**Imprisonment relative risk ratio**	**Solitary confinement by** **age 32 (%)**	**Solitary relative risk ratio**
*Men*				
White	2.82	1.00	1.35	1.00
Black	19.05	6.76	11.09	8.20
Latino	6.60	2.34	3.41	2.52
Other	0.62	0.22	0.29	0.22
Total	5.26	–	2.80	–
*Women*				
White	0.48	1.00	0.15	1.00
Black	0.83	1.72	0.40	2.75
Latina	0.42	0.88	0.16	1.10
Other	0.10	0.20	0.04	0.27
Total	0.48	–	0.17	–

We also find high rates and larger racial disparities for solitary confinement. Among black men in Pennsylvania born in the late 1980s, one in nine (11.1%) had been held in solitary confinement for at least 1 day by age 32. Nearly 60% of incarcerated black men in the birth cohort also spent time in solitary confinement. In comparison, 3.4% of Latino men and 1.4% of white men in the study birth cohort had been incarcerated in solitary confinement by their early 30s. The risk of solitary confinement by age 32 for black men is more than 8 times the risk for white men, and Latinos are 2.5 times as likely as white men to have been held in solitary confinement.

Cumulative risks of imprisonment and solitary confinement among women are significantly lower than among men. Men are about 10 times more likely to go to prison than women. Among all women in Pennsylvania, born 1986 to 1989, we estimate that one-half of 1% had been sent to prison by age 30. Among black women in the study birth cohort, 0.8% have been imprisoned by age 30, about twice the prevalence of imprisonment as for white and Latina women. Solitary confinement is also used less often among incarcerated women than incarcerated men. Nearly 0.2% of Pennsylvania women, born 1986 to 1989, have been in solitary confinement by age 32. The highest cumulative risk of solitary confinement is estimated for black women, whose rate of 0.4% is nearly three times the cumulative risk for white and Latina women.

Given the racial disparity in imprisonment and solitary confinement, how much of the disparity in solitary confinement results from high risks of imprisonment among black and Latino men and women versus high risks of solitary confinement once imprisoned? We decompose the racial and ethnic disparity in the cumulative risks of solitary confinement into components for the disparity in incarceration in the general population and disparity in solitary confinement, conditional on imprisonment (Table 4). For this decomposition, the total disparity between, say, black and white men is defined as the log cumulative risk for black men minus the log cumulative risk for white men. The total disparity in solitary confinement can be written as a function of the black-white disparity in the cumulative risk of incarceration in the population and the black-white disparity in solitary confinement among those who are in prison.

**Table 4.** Racial disparity in imprisonment and solitary confinement and decomposition results. Racial/ethnic disparities in cumulative risks of incarceration and solitary confinement reported as the difference of logs and relative risks, and decomposition results for racial/ethnic disparities in the cumulative risk of solitary confinement by gender in the Pennsylvania birth cohort, born 1986 to 1989.

	**Difference of logs**	**Relative risk**	**%**
*Men*			
Black-white disparity			
Incarceration	1.91	6.76	90.8
Solitary given incarceration	0.19	1.21	9.2
Total solitary	2.10	8.20	100.0
Latino-white disparity			
Incarceration	0.85	2.34	92.0
Solitary given incarceration	0.07	1.08	8.0
Total solitary	0.93	2.52	100.0
Black-Latino disparity			
Incarceration	1.06	2.88	89.9
Solitary given incarceration	0.12	1.13	10.1
Total solitary	1.18	3.25	100.0
*Women*			
Black-white disparity			
Incarceration	0.54	1.72	53.9
Solitary given incarceration	0.47	1.59	46.1
Total solitary	1.01	2.75	100.0
Latino-white disparity			
Incarceration	−0.13	0.88	−133.4
Solitary given incarceration	0.22	1.25	233.4
Total solitary	0.09	1.10	100.0
Black-Latino disparity			
Incarceration	0.67	1.95	73.1
Solitary given incarceration	0.25	1.28	26.9
Total solitary	0.92	2.50	100.0

Decomposing the disparity in solitary confinement shows that 90% of the relatively high rate of solitary confinement among black and Latino men is related to the disparity in incarceration in the population. The remaining 10% is related to the relatively high risk of solitary confinement among black and Latino men in prison. With less data for women, the results are more varied. However, a notable fraction of the risk of solitary confinement is related to the relatively high risk of solitary confinement in prison among incarcerated women of color.

**Fig. 1.**
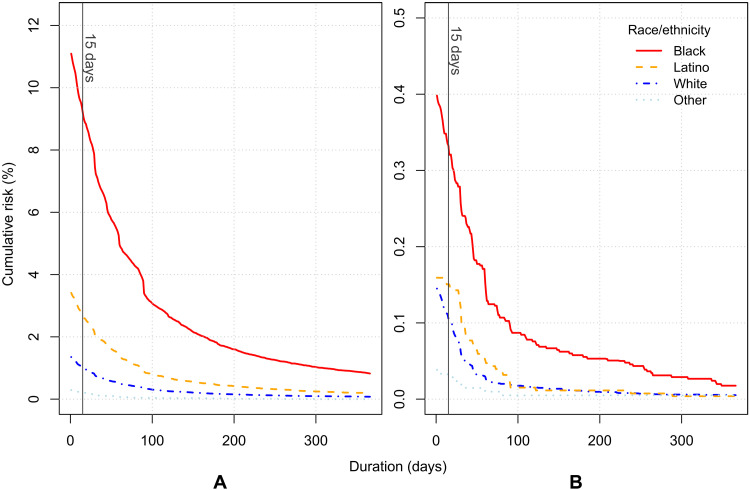
Solitary by duration. Cumulative risk at age 32 in Pennsylvania, by duration of solitary confinement in days and by race/ethnicity for men (**A**) and women (**B**).

Last, we report on the cumulative risk of solitary confinement for different minimum durations. Figure 1 shows the cumulative risk of solitary confinement from at least 1 day to more than 365 days for men (A) and women (B). The vertical line indicates 15 days of solitary confinement, the benchmark for prolonged solitary confinement designated by the United Nations. We estimate that 9% of all black men born between 1986 and 1989 in Pennsylvania have been incarcerated in solitary confinement for a period exceeding 15 days by age 32, compared to 2.7% of Latino men, and 1.1% of white men in the same birth cohort. For men incarcerated in solitary confinement, about 80% are thus held for longer than the United Nations limit on the minimum treatment of prisoners. Nearly 1 in 100 black men in Pennsylvania in the study cohort has been locked in solitary confinement for at least a full consecutive year by age 32, compared to a cumulative risk of 0.2% for Latino men and 0.08% for white men.

**Fig. 2.**
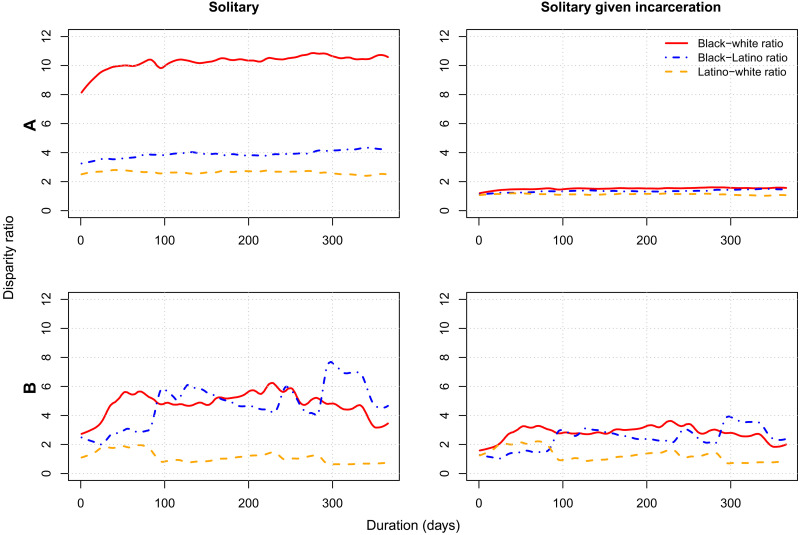
Racial/ethnic disparity by duration of solitary confinement. Smoothed relative cumulative risks of solitary confinement and solitary confinement given incarceration, for men (**A**) and women (**B**) by number of consecutive days in solitary confinement.

Examining racial and ethnic disparity across durations of solitary confinement, we find that black men are about 8.2 times more likely to spend at least a day in solitary confinement compared to white men by age 32 (Fig. 2). That disparity increases to 10.6 times for periods of solitary confinement of at least a year. The Latino-white ratios remain relatively stable across all durations of solitary confinement. The black-Latino disparity grows with duration, indicating that black men are disproportionately likely to experience long periods of solitary confinement. The relative risks of solitary confinement given incarceration follow a similar pattern to the overall solitary confinement disparity, increasing over longer durations for black men compared to white and Latino men. At all durations, the relative risk of solitary confinement in the population is much higher than the relative risk of solitary confinement given incarceration. Thus, most of the disparity in prolonged solitary confinement in the population results from the racial/ethnic disparity in incarceration rather than the disparity in treatment within the prison.

The pattern of increasing disparity is more varied for women because prolonged periods of solitary confinement are less common among all women (Fig. 2). The black-white disparity remains relatively high across all durations of solitary confinement for women, peaking at 220 days, where the black-white ratio is 6.3. The Latina-white disparity remains relatively low across durations and decreases for longer durations. Similar to the pattern observed for men, most of the disparity in prolonged solitary confinement results from the disparity in incarceration rather than the disparity in solitary given incarceration. However, disparity in treatment in prison, indicated by the relative risk of solitary confinement given incarceration, is larger for men than women.

## DISCUSSION

Among black men in Pennsylvania, born 1986 to 1989, one in nine has been locked in solitary confinement in state prison by age 32. The cumulative risk of solitary confinement for black men is three times higher than for Latino men and more than eight times higher than for white men. Although we find a similarly large racial disparity for women, the overall prevalence of solitary confinement is more than 90% lower than the prevalence for men.

Unusual by international standards, long periods of solitary greater than 15 days are also relatively common in Pennsylvania. Estimates indicate that 9% of all black men born 1986 to 1989 in Pennsylvania have spent at least 15 consecutive days, and almost 1 in every 100 has spent at least 1 year, in solitary confinement by age 32. Racial disparity in the population prevalence of the duration of solitary confinement persists through at least 1 year of solitary confinement, with black men more likely to be held for long stays than any other racial/ethnic group.

A decomposition shows that the overrepresentation of black men in solitary confinement in the Pennsylvania population is primarily the result of the overall racial disparity in incarceration. Roughly 10% of the black-white cumulative risk of solitary confinement is related to the racial disparity in solitary confinement inside prisons. The decomposition results suggest that there may be greater potential to reduce the relatively high exposure of black men to solitary confinement by lowering the prevalence of, and disparity in, incarceration in the population. The racial disparity that black men and women experience increases for longer durations of solitary confinement, and this, too, mostly results from the racial disparity in imprisonment.

The current analysis is subject to several limitations. First, the findings apply only to Pennsylvania, and it is unclear whether similar rates and disparities would be found in other states. Because solitary confinement is used in a similar way to other jurisdictions (*31*) and rates of solitary confinement in the state mirror national levels (*5*), we believe that similar patterns would likely be found elsewhere. Still, racial disparities in imprisonment vary across states (*28*). States with relatively high disparities in imprisonment would likely have higher disparities in the risk of solitary confinement than those reported here. Second, the data only allow us to explore prevalence from ages 18 to 32 and only within state prisons. Without data on solitary confinement in other types of incarceration such as jails, and more data across the life course, the results underestimate the population prevalence of solitary confinement. Third, stratifying the analysis by additional demographic measures such as education would likely yield even greater disparity in the risk of solitary confinement because of the high rate of imprisonment among people with little schooling. In particular, pervasive imprisonment among black men with very little schooling (*30*) may be matched by pervasive solitary confinement in this same segment of the population.

The evidence indicates that a high and disparate rate of imprisonment is closely associated with high rates of population-level exposure to solitary confinement among black men. Because solitary confinement has harmful effects on health and well-being, and federal courts have scrutinized conditions of extreme isolation, the pattern of imprisonment itself may have a social impact, threatening public health and collective security against cruel and unusual punishment guaranteed by the Constitution. Although efforts to improve prison conditions may reduce the harms of incarceration, our evidence indicates that large reductions in black men’s absolute and relative exposure to solitary confinement will depend on reducing the general level and racial disparity of imprisonment.

## MATERIALS AND METHODS

Pennsylvania has 23 state prisons, with 2 designated for women. Prison conditions vary widely. The oldest prison in the system was built in 1889, and 16 prisons opened after 1990 with the newest facility having opened in 2018. All have solitary confinement units. In solitary confinement—whether for disciplinary or administrative custody—incarcerated people are provided with basic supplies for clothing, bedding, and hygiene, and staff provide food through a slot in the door. Typical cells are about 3 meters by 2.5 meters and include one or two fixed bunk beds, a toilet, and sink. These conditions are similar to those found nationwide (*3*, *5*, *31*).

Estimates of the cumulative risks of imprisonment and solitary confinement are based on census data, vital statistics, and prison administrative records. The core data file records all prison admissions and discharges from 2007 to 2016 in the state, with data on those already admitted continuing through February 2018. The dataset includes demographic and prison misconduct information and detailed records on the incidence and dates of solitary confinement. We operationalize solitary confinement and duration using a combination of misconduct charge records and the admission and release dates that indicate when an incarcerated person entered and left disciplinary or administrative custody. A solitary confinement stay in this analysis includes all records where an individual was held in solitary confinement for at least 1 day, thus avoiding potential overinflation from temporary holds for transfers or court appearances. Our analysis is confined to individuals born 1986 to 1989, allowing us to observe first-time imprisonment and solitary confinement in Pennsylvania. With data from 2007 to 2018, the birth cohort ages from 18 to 32. Time-invariant person-level identification numbers allow us to identify individuals across prison terms, ensuring that first-imprisonment and first-solitary risk estimates reflect the experiences of individuals, rather than distinct prison admissions for the same person.

To calculate cumulative risks, we begin by estimating the age-specific risks of imprisonment and solitary confinement. At age *a* = 18, 19,…32, the age-specific risk of solitary confinement, for example, is estimated as the count of the number of people sent to solitary confinement for the first time, *S_a_*, divided by the population at risk of first-time solitary confinement, ${\hat{P}}_{a}$,

$$r_{a}=S_{a}/{\hat{P}}_{a}$$ (1)

The at-risk population is the observed population, *P_a_*, minus all those who have been in solitary confinement and survived to age *a*, where survivors are those still living who have not left the state. Population counts by gender, race/ethnicity, and cohort are obtained from the 2010 U.S. Decennial Census and American Community Survey (ACS). To adjust for migration and mortality, we calculate annual Pennsylvania migration rates from the ACS and age-specific mortality by race/ethnicity and gender from the CDC WONDER Database. Following the 1986–1989 birth cohort from age 18, we calculate age-specific risks for each year of age, the cumulative risk of imprisonment to age 30 in 2016, and the cumulative risk of solitary confinement to age 32 in 2018.

To estimate the cumulative risk, the age-specific risks, *r_a_*, are used to expose a hypothetical population of size, ${\tilde{P}}_{a}$, at risk of being imprisoned and of going to solitary confinement for the first time. We set the baseline population, ${\tilde{P}}_{18}$, called the radix in demographic life table analysis, to 100,000 (*29*). Exposing a population to age-specific risks yields the number going to solitary confinement, ${\tilde{S}}_{a}=r_{a}{\tilde{P}}_{a}$, at each year of age. The cumulative risk is given by the total ever in solitary confinement as a proportion of the population. For the cumulative risk of solitary confinement

$$c=\frac{\sum_{a=18}^{32}{\tilde{S}}_{a}}{{\tilde{P}}_{18}}$$ (2)

We also apply this method to estimate the cumulative risk of imprisonment to age 30 and the cumulative risk of solitary confinement to age 32 by the duration of stay in solitary confinement for minimum durations up to a year or more. For the duration calculations, we define first solitary confinement as the first solitary stint that meets the duration threshold. For example, if an individual is held in solitary for 5 days at age 18 and for 15 days at age 20, their first time in solitary confinement for 1 day or more would occur at age 18; for 15 days or more would occur at age 20; and for longer durations, such as 30 days or more, they would have no qualifying solitary confinement experience.

Table 5 reports descriptive statistics for the racial/ethnic composition of the 1986–1989 birth cohort for the total Pennsylvania state birth cohort population, the prison cohort population, and the cohort population that has spent at least 1 day in solitary confinement. The prison-admitted birth cohort is 91% male, and the racial composition of the prison population varies by gender. Over half of the prison admissions for men in the study cohort are black or Latino, compared to under 30% among prison admissions for women. Black men in this birth cohort are overrepresented in the prison population and solitary confinement. While black men are only 12% of the state cohort population, they make up 52% of the total cohort population of men in solitary confinement.

**Table 5.** Population race/ethnicity compositions. Percentage distribution of race/ethnicity of a Pennsylvania birth cohort, born 1986 to 1989, by gender for the total state census population, the cohort admitted to prison by age 30 (2007–2016), and the cohort held in solitary confinement by age 32 (2007–2018).

	**White (%)**	**Black (%)**	**Latino (%)**	**Other (%)**	**Sample size (*N*)**
*Men*					
State population (2010)	74.2	12.4	8.0	5.3	345,222
Prison population (2007–2016)	40.6	47.8	10.9	0.7	16,906
Solitary population (2007–2018)	36.3	52.4	10.7	0.6	9061
*Women*					
State population (2010)	74.3	12.9	7.3	5.6	336,982
Prison population (2007–2016)	70.5	22.0	6.3	1.1	1626
Solitary population (2007–2018)	61.6	30.1	6.9	1.4	562

Table 6 reports descriptive statistics for the Pennsylvania administrative data for the 1986–1989 birth cohort’s exposure to solitary confinement. Over half of the men in the study cohort who have been to prison have also been incarcerated in solitary confinement. Nearly 60% of black men have been in solitary confinement for at least 1 day, the highest solitary confinement rate among all race and ethnic groups. Among those who have been in solitary confinement, the median length of stay is 30 days. The median total number of days that an individual in this birth cohort spends in solitary confinement during the study period is 63 days, with black and Latino men spending more days overall in solitary confinement than white men or men of another race. Solitary confinement is less common for women in prison than it is for incarcerated men, and the women spend less time in solitary confinement than men. Black and Latina women stay longer in solitary confinement, with black women spending more than twice as many total days in solitary confinement during the study period as white women.

**Table 6.** Solitary confinement exposure statistics. Solitary confinement incarceration characteristics of a Pennsylvania prison admission cohort, born 1986 to 1989, by gender and race/ethnicity.

	**Ever in solitary** **confinement (%)**	**Median** **average time** **in solitary** **confinement** **(days)**	**Median** **cumulative** **time in solitary** **confinement** **(days)**
*Men*			
White	47.9	26.5	48.0
Black	58.7	33.0	77.0
Latino	52.9	29.0	60.0
Other	47.4	26.0	30.0
Total	53.6	30.0	63.0
Sample size (*N*)	16,906	9061	9061
*Women*			
White	30.2	20.8	30.0
Black	47.2	29.7	65.0
Latina	37.9	31.0	45.0
Other	44.4	24.0	41.5
Total	34.6	24.2	41.0
Sample size (*N*)	1626	562	562

Racial disparity in the cumulative risk of solitary confinement can be decomposed with estimates of the cumulative risk of imprisonment (see Table 4). Calculations for the decomposition of racial disparity require cumulative risks of imprisonment and solitary confinement for each of the four race/ethnicity groups, *r* = *B*, *W*, *L*, *O*, for black, white, Latino, and other. The cumulative risk of solitary confinement, interpreted as a probability, π*_Sr_* = *p*(*S_r_*), is a function of the probability of incarceration, π*_Ir_* = *p*(*I_r_*), and the probability of solitary given incarceration, π_*S*∣*Ir*_ = *p*(*S_r_*∣*I_r_*)

$$\pi_{\mathrm{Sr}}=\pi_{S|\mathrm{Ir}}\times \pi_{\mathrm{Ir}}$$ (3)

The black-white disparity, for example, can be written

$$\text{log}\;\pi_{\mathrm{SB}}-\text{log}\;\pi_{\mathrm{SW}}=(\text{log}\;\pi_{\mathrm{IB}}-\text{log}\;\pi_{\mathrm{IW}})+(\text{log}\;\pi_{S|\mathrm{IB}}-\text{log}\;\pi_{S|\mathrm{IW}})$$ (4)

The first term on the right-hand side, logπ*_IB_* − log π*_IW_*, is the disparity in the cumulative risk of incarceration. The second term, logπ_*S*∣*IB*_ − log π_*S*∣*IW*_, is the disparity in solitary confinement given incarceration. The calculation of cumulative risks yields estimates of π*_Ir_* and π*_Sr_*, which can be used to calculate the third decomposition quantity, π_*S*∣*Ir*_ = π*_Sr_*/π*_Ir_*.

Because solitary confinement is experienced with some lag following incarceration, estimates of racial disparity are based on the prevalence of imprisonment by age 30 but the prevalence of solitary confinement by age 32. For the observed birth cohort, the first experience of solitary confinement occurs, on average, 1.3 years after first incarceration. Of those who are held in solitary confinement, 77% have been placed in solitary within 2 years of their prison admission, and thus, a 2-year observation lag provides a good estimate of the experienced lag time to first solitary stint.
